# Scientific Medication and Specific Medicines

**Published:** 1888-04

**Authors:** 


SCIENTIFIC MEDICATION AND SPECIFIC MEDICINES. By Dr. John M. Scudder. Twelfth edition. Cincinnati. 1888.

That such a worthless book as this can go into a twelfth edition is a sad commentary on the intelligence of physicians.

